# Reliability and validity of Oswestry Disability Index among patients undergoing lumbar spinal surgery

**DOI:** 10.1186/s12893-023-02307-w

**Published:** 2024-01-03

**Authors:** Konsta Koivunen, Sara Widbom-Kolhanen, Katri Pernaa, Jari Arokoski, Mikhail Saltychev

**Affiliations:** 1 Clinical Division, Turku University Hospital and University of Turku, Turku, 20540 Finland; 2 Department of Surgery, Satasairaala Hospital, Pori, Finland; 3 Department of Orthopedics, Turku University Hospital and University of Turku, Turku, Finland; 4 Department of Physical and Rehabilitation Medicine, Helsinki University Hospital and Helsinki University, Turku, Finland; 5 Department of Physical and Rehabilitation Medicine, Turku University Hospital and University of Turku, Turku, Finland

**Keywords:** Patient reported outcome measures, Pain measurement, Psychometrics, Disability evaluation, Surveys and questionnaires, Low back pain, Orthopedic procedures

## Abstract

**Background:**

The objective of this study was to explore the internal consistency and factor structure of the Oswestry Disability Index among patients undergoing spinal surgery. The sample consisted of 1,515 patients who underwent lumbar spinal surgery at a university hospital between 2018 and 2021.

**Methods:**

The patients responded to the Oswestry Disability Index within 2 months before surgery. Cronbach’s alpha was used to assess the internal consistency. The factor structure was evaluated using exploratory and confirmatory factor analyses.

**Results:**

The average age of 1,515 patients was 58.5 (SD 15.8) years and 53% were women. The mean ODI score was 43.4% (SD 17.4%). Of the patients, 68% underwent microsurgical excision of the lumbar intervertebral disc displacement or decompression of the lumbar nerve roots. The internal consistency of the Oswestry Disability Index was found to be good, with an alpha of 0.87 (95% CL 0.86 to 0.88). Exploratory factor analysis resulted in unidimensional structure. Item loadings on this retained factor were moderate to substantial for all 10 items. One-factor confirmatory factor analysis model demonstrated an acceptable fit. The correlations between the main factor “disability” and the individual items varied from moderate (0.44) to substantial (0.76). The highest correlations were observed for items “traveling”, “personal care”, and “social life”. The lowest correlations were observed for the item “standing”.

**Conclusions:**

The Oswestry Disability Index is a unidimensional and internally consistent scale that can be used to assess the severity of disability in patients undergoing lumbar spinal surgery. In the studied population, “traveling,” “social life,” “sex life” and “personal care” were the most important items to define the severity of disability, while “walking” and “standing” were the least important items. The generalizability of the results might be affected by the heterogeneity and modest size of the studied cohort.

**Trial registration:**

Not applicable.

**Supplementary Information:**

The online version contains supplementary material available at 10.1186/s12893-023-02307-w.

## Context

The Oswestry Disability Index has been used worldwide for assessing disability caused by low back pain. This study investigated how well the Oswestry Disability Index performs amongst patients undergoing lumbar spinal surgery.

**Supplementary Information:**

The online version contains supplementary material available at 10.1186/s12893-023-02307-w.

## Introduction

At least 200 patient-reported outcome measures (PROMs) are used for different purposes in patients with spinal complaints, including those undergoing spinal surgery [1]. During the last four decades, the Oswestry Disability Index (ODI) has become a well-researched gold standard for assessing the severity of disability caused by back pain [2–6]. The ODI is the most commonly used PROM to assess the limitations of functioning in spinal conditions treated either operatively or conservatively [7]. In 2020, a review of different measures of disability caused by low back pain suggested that the Roland Morris Disability Questionnaire and the ODI are the most widely accepted scales for the task among people with spinal disorders such as herniated intervertebral discs, spinal infection, spondylosis, and spondylolisthesis, among many others [8]. The ODI has been translated into numerous languages, and modified versions have been suggested. However, the developer of the ODI Dr. Jeremy Fairbank stated that none of the modifications made to the ODI have proven to be better than the original one [9].

The internal consistency of the ODI has usually been found to be good, with an alpha up to 0.90 or even higher [10]. This included the Finnish version of the ODI, which was also used in the present study [11]. Other studies have reported a slightly lower alpha of 0.7 to 0.8 [12, 13]. A review of 16 studies reported an overall good internal consistency of the ODI, with a Cronbach’s alpha of approximately 0.9 [4].

Many previous studies have found that ODI is unidimensional [14–16]. However, other studies reported a two-factor structure [11, 12, 17, 18]. For example, exploratory factor analysis (EFA) of 60,000 people undergoing spinal surgery resulted in a two-factor structure of the ODI [12]. Another study employing the same ODI translation as that used in the present study observed a two-factor structure [11]. Confirmatory factor analysis (CFA) among 35,000 patients has resulted in substantial variability across the loadings of the ODI items on a common “disability” factor varying from 0.53 to 0.81 [19].

This variability in the alpha and factor structures of the ODI may be explained by differences in the settings and studied populations. For example, a population predominated by a particular age group or sex may demonstrate deviant scores concerning the ODI item “sex life” [20], and people undergoing spinal surgery may score higher on the ODI than conservatively treated patients [16]. In addition, differences between samples in the overall disability level may affect the internal consistency of the ODI, which has been reported to have a better discriminative ability among people with more severe disability signaling owing to the existence of the floor effect [21]. A review comparing the ODI with the Roland-Morris Disability Questionnaire reported that both scales might have different internal consistencies across diverse populations and translations, calling for further research [22]. In 2018, a review of different PROMs in lumbar spinal surgery stated that while the ODI has been validated in diverse populations consisting of people with back pain, it has not been validated in patients undergoing lumbar spinal surgery [3].

Although the ODI is an overall well-studied scale, there is still some uncertainty regarding its reliability and validity when applied to a particular population of patients undergoing spinal surgery. The objective of this study was to explore the internal consistency and factor structure of the ODI in patients undergoing spinal surgery.

## Methods

Data were obtained from an ongoing register-based study of patients undergoing cervical, thoracic, or lumbar spinal surgery (CTL Study) at a university hospital. Patients responded to a survey ≤ 2 months prior to surgery. The survey contained questions on demographics and disability severity. The present study used data on patients who underwent lumbar spinal surgery of any kind between June 21, 2018, and August 17, 2021 (Table 1). Patients who had undergone more than one procedure during follow-up were excluded. According to the ethics board of the university hospital district, this register-based study does not require specific approval.

**Table 1:** Distribution of surgery codes according to the Nordic Classification of Surgical Procedures (NCSP) version 1.15

Procedure code	Procedure	n	%
ABC16	Microsurgical excision of lumbar intervertebral disc displacement	439	29
ABC36	Decompression of lumbar nerve roots	297	20
NAG62	Posterior fusion of lumbar spine with fixation, 2–3 vertebrae	282	19
ABC56	Decompression of lumbar spinal canal and nerve roots	271	18
NAG66	Posterior interbody fusion of lumbar spine, 2 vertebrae	71	5
NAG63	Posterior fusion of lumbar spine with fixation > 3 vertebrae	54	4
ABC66	Decompression of lumbar spinal channel	48	3
ABC26	Open discectomy of lumbar spine	34	2
NAG61	Posterior fusion of lumbar spine without fixation	12	1
ABC07	Percutaneous lumbar endoscopic discectomy	4	0
NAG67	Posterior interbody fusion of lumbar spine > 2 vertebrae	2	0
NAJ32	Posterior reduction of fracture of lumbar spine	1	0

Age was defined as full years at the time of surgery. Body mass index (BMI) was defined as body weight divided by height squared and was expressed in kg/m^2^. The preoperative pain duration was defined as < 6 weeks, 6–12 weeks, 3–12 months and > 12 months before surgery. Back pain intensity was assessed using a visual analog scale varying from 0 to 100 points, with 0 indicating ‘no pain’ and 100 indicating the most likely pain.

The ODI is a questionnaire containing 10 items covering disability caused by low back pain. Each item was assessed on a six-level ordinal scale with ‘0’ describing ‘no limitation’ and ‘5’ describing ‘extreme limitation or an inability to function’. The total score is a percentage calculated by the sum of all answers divided by 50 (the maximum possible number of points) and multiplied by 100 as follows: ‘Total score = (∑item scores/50) x 100’. The equation was adjusted when the responses to one or more items were missing. The missing responses were not imputed, but considered ‘missing’. E.g., if one item response was missing, then the sum was calculated as (∑item scores/45) x 100. A score of 0% represents the highest possible level of functioning and independence, whereas a score of 100% represents the lowest possible level of functioning with total dependence. The Finnish version of the ODI was used [11]. The variables collected in this study were the same as those in the Finspine registry [23]. The methods of gathering these register-based data have previously been described by the present research team [24, 25].

### Statistical analysis

The descriptive characteristics of the sample were presented as absolute numbers and percentage or as means and standard deviations (SD).

#### Internal consistency

The internal consistency of the ODI was assessed using Cronbach’s alpha. Alpha ≥ 0.9 was considered excellent; ≥ 0.8, good ≥ 0.7, acceptable, ≥ 0.6, questionable; ≥ 0.5, poor and < 0.5 was considered unacceptable [26]. A sensitivity test was performed by excluding each item at a time. While, there is no consensus on a smallest reliable sample size for a factor analysis, it has often been suggested that a sufficient sample size for a factor analysis may be between 3 and 20 times the number of test items [27]. The present study used these ‘rules of thumb’, instead of probably more efficient power calculations like Satora-Sarris or Monte-Carlo method or Bayesian approach [27, 28].

#### Exploratory factor analysis (EFA)

Data were randomly split into two equal parts and adjusted for age and sex. First of these two parts (n = 759) were used in the EFA to approximate the construct structure of the ODI. The other half (n = 756) was used for CFA. The goal was to determine whether the ODI measured only one latent trait (e.g., disability) or if there were other possible significant latent variables affecting the results. The results were analyzed numerically and graphically. EFA (principal factors) was applied with a minimum eigenvalue for retention set at > 1.0 (Kaiser’s rule) [29]. Retained and excluded factors were also explored visually on a scree plot accompanied by parallel analysis.

#### Confirmatory factor analysis (CFA)

This study employed CFA to verify the construct structure of the ODI, as seen in an exploratory factor analysis. CFA extends the abilities of EFA to measure errors in a model. The estimation procedure uses the maximum likelihood method, considering the covariances supplied as the input to be unbiased. For simplicity, the estimates were reported in a standardized form as correlation coefficients. A correlation < 0.2 was considered poor, from 0.21 to 0.4 fair, from 0.41 to 0.6 moderate, from 0.61 to 0.8 substantial, and > 0.8 perfect [30].

**Fig. 1:**
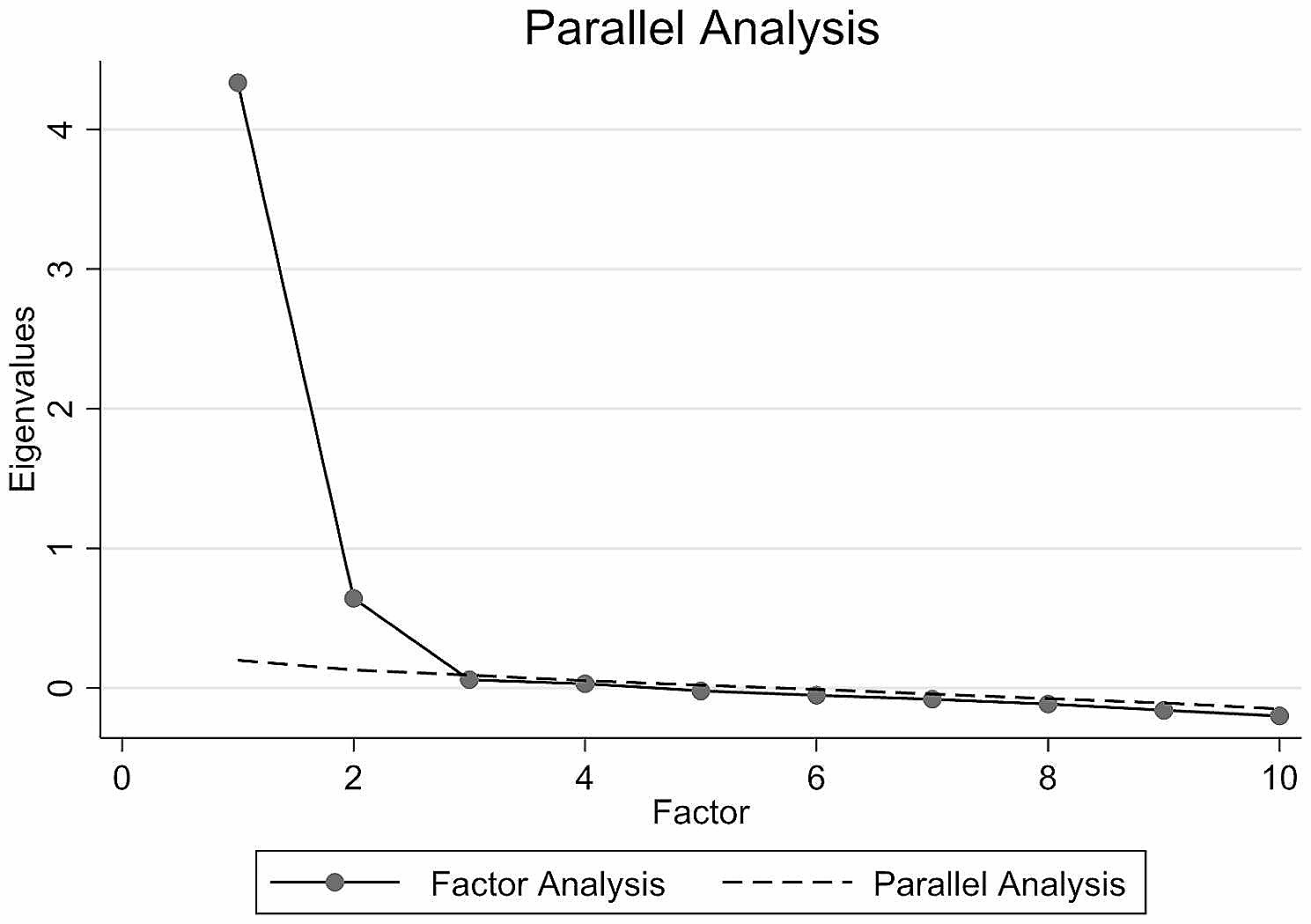
Scree plot (n = 759)

To assess how well the model matched the observed data, the root mean square error of approximation (RMSEA) was used. First, the model fit was tested by assuming that there were no covariances between unique factors. After that, the modification indices suggested by the software were used to add covariances between factors (double-headed arrows in Fig. 1) one at a time, each time testing the lower 90% confidence limit (90% CL) of RMSEA closeness to 0.05 and upper 90% CL closeness to 0.10. The probability of RMSEA being ≤ 0.05 was also reported. Every insertion was considered plausible if it made logical sense and did not violate the assumption that the common and unique factors were uncorrelated. After achieving an acceptable RMSE value, no further covariances were imputed, and the overall goodness of fit was assessed using a chi-square test for the difference between the model used and a saturated model (a model with a theoretically perfect fit). The results were accompanied by Akaike’s information criterion, Bayesian information criterion, comparative fit index, Tucker–Lewis index, standardized root mean squared residual, and coefficient of determination.

Due to the heterogeneity in surgical procedures, to illuminate whether there was a difference between ODI performance by surgical procedure, the subgroup analysis was performed dividing the sample into two groups: ‘discectomy’ group (‘ABC07’, ‘ABC16’, ‘ABC26’) and ‘decompression’ group (‘ABC36’, ‘ABC56’, ‘ABC66’, ‘NAG61’, ‘NAG62’, ‘NAG63’, ‘NAG66’, ‘NAG67’). The alpha was calculated, and the scree plot for the EFA and the path diagram for the CFA (assuming the same covariance between measurement errors) analyses were drawn for each group.

All analyses were conducted using Stata/IC Statistical Software Release 17. College Station (StataCorp LP, TX, USA).

## Results

Data were available for 1,515 patients (Table 2). Their average age was 58.5 (SD 15.8) years. Of these, 809 (53%) were women, and 706 (47%) were men. The mean ODI score was 43.4% (SD 17.4%). Of these, 68% underwent microsurgical excision of the lumbar intervertebral disc displacement or decompression of the lumbar nerve roots (Table 1). The most frequent reason for the surgery was “M48 spinal stenosis” (38%) (Table 3).

**Table 2:** Descriptive characteristic of the sample

Variables	Mean	Standard deviation
Age, years	58.5	15.8
Body mass index (BMI), kg/m^2^	28.5	5.0
Back pain severity, points	60.4	26.8
Oswestry disability Index, points	43.4	17.4
Pain duration before surgery	N	%
< 6 weeks	118	8
6–12 weeks	196	13
3–12 months	487	33
> 12 months	681	46

**Table 3:** Distribution of main diagnoses codes according to the International Statistical Classification of Diseases and Related Health Problems, 10th Edition (ICD-10)

Diagnosis (ICD-10)	Dg title	n	%
M48	Spinal stenosis	570	38
M51	Intervertebral disc disorders	315	21
G55	Nerve root and plexus compressions	271	18
M43	Deforming dorsopathies	189	12
M47	Spondylosis	86	6
M41	Scoliosis	27	2
M71	Bursopathies	18	1
M53	Dorsopathies	15	1
M80	Osteoporotic fracture	9	1
Others		15	1
Total		1,515	100

### Internal consistency

The Cronbach’s alpha was good at 0.87 (95% CL 0.86 to 0.88) (Table 4). All items demonstrated good item-test and item-rest correlations. In addition, excluding one item at a time did not improve the alpha.

**Table 4:** Internal consistency of ODI (Cronbach’s alpha), n = 1,515

Item		n	Sign	Item-test correlation	Item-rest correlation	Average interitem covariance	Alpha
Item 1	Pain intensity	1515	+	0.66	0.58	0.67	0.86
Item 2	Personal care	1515	+	0.74	0.68	0.65	0.85
Item 3	Lifting	1515	+	0.70	0.62	0.65	0.85
Item 4	Walking	1515	+	0.61	0.50	0.68	0.86
Item 5	Sitting	1515	+	0.62	0.52	0.68	0.86
Item 6	Standing	1515	+	0.59	0.49	0.68	0.86
Item 7	Sleeping	1515	+	0.59	0.50	0.70	0.86
Item 8	Sex life	1515	+	0.75	0.63	0.59	0.86
Item 9	Social life	1515	+	0.76	0.68	0.63	0.85
Item 10	Travelling	1515	+	0.78	0.71	0.62	0.85
Total score						0.65	0.87^a^

^a^95% CI 0.86 to 0.88

### Exploratory factor analysis (EFA)

The EFA demonstrates the unidimensionality of the ODI. A single factor with an eigenvalue of 4.02 was retained (Tables 5 and 6; Fig. 1). Item loadings on this retained factor were moderate to substantial for all ten items, varying from 0.52 to 0.76. The level of unique variance varied from 0.43 to 0.73.

**Table 5:** Exploratory factor analysis – item loadings (n = 759)

Items		Factor 1	Uniqueness
Item 1	Pain intensity	0.60	0.64
Item 2	Personal care	0.70	0.50
Item 3	Lifting	0.65	0.58
Item 4	Walking	0.55	0.70
Item 5	Sitting	0.58	0.67
Item 6	Standing	0.52	0.73
Item 7	Sleeping	0.53	0.72
Item 8	Sex life	0.68	0.53
Item 9	Social life	0.72	0.48
Item 10	Travelling	0.76	0.43

**Table 6:** Parallel analysis (n = 759)

Factors	Eigenvalues	Eigenvalues averaged over 10 replications	Difference
1	4.02	0.21	3.82
2	0.63	0.15	0.49
3	0.07	0.09	-0.02
4	0.05	0.06	-0.01
5	-0.01	0.02	-0.03
6	-0.06	-0.01	-0.05
7	-0.09	-0.04	-0.05
8	-0.13	-0.08	-0.04
9	-0.17	-0.12	-0.06
10	-0.20	-0.15	-0.05

### Confirmatory factor analysis (CFA)

**Fig. 2:**
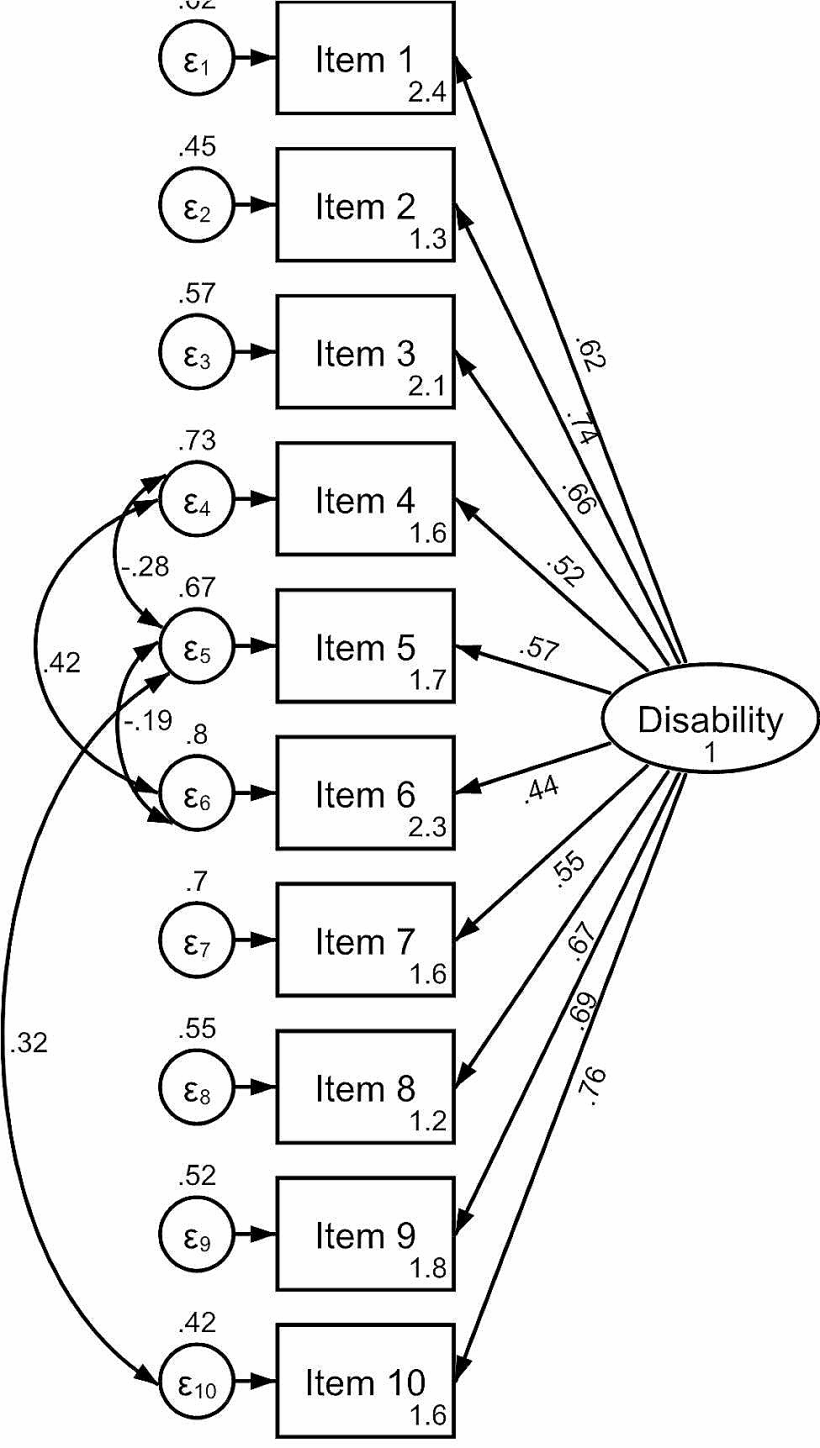
Path diagram of confirmatory factor analysis (n = 756)

The 1-factor model of the CFA model demonstrated an acceptable fit (Table 7). The covariances of measurement errors were imputed for items “walking”, “sitting”, “standing”, and “traveling” (Fig. 2). The correlations between the main factor “disability” and the individual items varied from moderate (0.44) to substantial (0.76). The highest correlations were observed for items “traveling” (0.76), “personal care” (0.74), and “social life” (0.69). The lowest correlations were observed for the item “standing” (0.44).

**Table 7:** Confirmatory factor analysis – goodness of fit (n = 756)

Fit statistic	Value
Likelihood ratio	98.154
chi2_bs(45)	2828.010
p > chi^2^	0.000
**Population error**	
Root mean squared error of approximation (RMSEA)	0.053
RMSEA 90% CI, lower bound	0.042
RMSEA 90% CI, upper bound	0.066
Probability RMSEA < = 0.05	0.298
**Information criteria**	
Akaike’s information criterion (AIC)	22320.515
Bayesian information criterion (BIC)	22478.003
**Baseline comparison**	
Comparative fit index (CFI)	0.976
Tucker-Lewis index (TLI)	0.965
**Size of residuals**	
Standardized root mean squared residual (SRMR)	0.034
Coefficient of determination (CD)	0.874

### Subgroup analysis

The subgroup analysis was performed dividing the sample into two groups: ‘discectomy’ group (n = 477 [32%]) and ‘decompression’ group (n = 1,037 [68%]). The alpha was 0.88 (95% 0.86 to 0.90) for the “discectomy” group, and 0.86 (95% CI 0.85 to 0.88) for the “decompression” group. The scree plot for the EFA and the path diagram for the CFA were very similar for all the models (Supplement 1).

## Discussion

This observational register-based study investigated the internal consistency and factor structure of the ODI in 1,515 patients undergoing lumbar spinal surgery. The internal consistency of the scale was good. All items were found to have good item-test and item-rest correlations, and it seemed that excluding any single item from the questionnaire would not improve alpha. The EFA found that the ODI was unidimensional. CFA was conducted assuming a one-factor structure. The highest correlations between items and a common factor were found for items “traveling”, “social life”, “sex life”, and “personal care”. The lowest correlation was found for the item “standing”.

These results are in line with previous research that reported good internal consistency of the ODI [4, 10, 11, 16, 21]. This includes a study by Selva-Sevilla et al., which reported an alpha over 0.9 among patients with previous lumbar spinal surgery [10]. In addition, a review by Sheahan et al. found an alpha close to 0.9 in all 16 included studies [4]. Pekkanen et al., using the same translation of the ODI as in a recent study, also reported an alpha value close to 0.9 [11]. These results were similar to those of earlier reports on the unidimensionality of the ODI [14–16, 19, 21, 31]. A study of 35,000 patients with degenerative disease of the intervertebral disc observed a one-factor structure of the ODI and factor loadings between 0.58 and 0.81, which is similar to the present findings [19]. Similar loadings between 0.59 and 0.77 have been reported for the Italian translation of the ODI [31]. In line with the present results, a similar factor structure of the ODI was observed when applying the CFA [19].

The unidimensionality of the ODI seen in this study contradicts some previous observations. Multiple studies have reported the two-factor structure of the ODI [11, 12, 17, 18]. A study among 60,000 patients undergoing spinal surgery observed the two-factor structure of the ODI when applying both exploratory and confirmatory factor analyses – the first factor included items “lifting”, “walking”, “standing”, “sex life” and “social life”, while the second factor contained items “pain intensity”, “personal care”, “sitting”, “sleeping” and “traveling” [12]. While some of previous studies have been conducted on larger samples, this fact might hardly explain the diversity in the results as the present sample size should be sufficient for exploratory factor analysis, as previously suggested [27]. While, there is no consensus on a smallest reliable sample size for a factor analysis, it has often been suggested that a sufficient sample size for a factor analysis may be between 3 and 20 times the number of test items. The difference between that report and the present findings might be related to the fact that more than half of the patients in that sample had significant comorbidities such as diabetes or arthritis [32]. Unfortunately, data on comorbidities were not available in the present register. Pekkanen et al. used the same translation as that used in the present study, reporting a two-factor structure, defining two factors as characterizing the activities of daily living and social life and another describing pain and activities in an upright position [11]. The difference from the present findings might be related to the small sample size of that study, leading to insufficient study power to conduct a factor analysis. Moreover, small samples might be the reason for observing the two-factor structure of the ODI when validating Arabic translations of the scale [17, 18].

In line with this study, the Dutch version of the ODI demonstrated high correlations between a common factor and items “traveling”, “social life”, and “sex life” [15]. Similar results were observed in previous studies [19, 21]. Previous research has suggested that item “social life” might have the greatest impact on a common factor understood as health-related quality of life [33].

The generalizability of these results may be affected by several factors. The study was conducted in a single, highly specialized university clinic; therefore, the results might differ in primary care. In addition, the results might be affected by particular age and gender distributions or the average level of disability; however, their effects were outside the scope of this study. There were a variety of symptoms, surgeries, and patient groups in the sample. For example, patients with a herniated disc tend to be younger and healthier than patients with spinal stenosis, who tend to be older and whose level of functioning can be low even without a spinal disorder. In the studied cohort, almost half of the respondents had a diagnosis of spinal stenosis, a clinical entity that is often not associated with severe pain, but rather with sensory loss and focal weakness motor. This fact may distort the generalizability of the results, as one of the criticisms of the ODI has been its heavy reliance on pain. The reason for “walking” and “standing”, two common complaints among patients with back pain, were found to be the two least important items in the confirmatory factor analysis was unclear. Due to a relatively modest sample size, any sub-sample analysis, which could clear out that phenomenon, was considered not advisable.

Further research may reveal the stability of the ODI psychometrics before and after surgery. For example, after surgery, pain and stiffness may affect responses to the ODI items. In addition, satisfaction with the results of surgery may affect the responses to particular items but not to others. There is also a need to investigate whether different comorbidities or prior spinal conditions affect the psychometric properties of the ODI. Rasch or item response theory analyses of similar populations are needed. Additionally, it is possible that there are some subgroups within the population of people undergoing spinal surgery that may demonstrate different patterns of the psychometrics of the ODI. Further research should focus on defining these groups.

There are several important clinical implications of these results. The good internal consistency of the ODI means that, in the studied population, all 10 items measure the same latent variable – disability caused by back pain. This is confirmed by the unidimensionality observed by a factor analysis. A unidimensional and internally consistent scale produces a composite score, which is comparable among different respondents. Thus, in a population that is similar to one studied here (people waiting for a lumbar spine surgery due to heterogenic reasons), the ODI composite score will describe reliably and comparatively the severity of disability. In addition to that, the present study completed the EFA with the CFA, which sort of ranked the importance of the ODI items, as perceived by the respondents, in this particular population. It seems that some items may be more relevant to the respondents than others. Because of that, while composite score is a good way to describe disability level on a group level, creating a functional profile of an individual patient could describe their disability severity with more precision. Such functional profile should be based on the scores obtained from the ODI individual items and it could be presented in a numerical or graphical form. In other words, the results suggest that the ODI is a reliable and valid scale in a heterogenic population of people expecting their spine surgery, and it is able to describe disability severity from two directions – as a composite score for a quick assessment or as a functional profile containing individual item scores for a more thorough evaluation on a personal level.

## Conclusions

The ODI is a unidimensional and internally consistent scale that can be used to assess the severity of disability in patients undergoing lumbar spinal surgery. In the studied population, “traveling”, “social life”, “sex life” and “personal care” were the most important items to define the severity of disability, while “walking” and “standing” were the least important items. The generalizability of the results might be affected by the heterogeneity and modest size of the studied cohort.

### Electronic supplementary material

Below is the link to the electronic supplementary material.

**Supplementary Material 1**. Factor analysis by surgery types - scree plots and path diagrams

## Data Availability

The data are available on a reasonable request from Mikhail Saltychev, Mikhail.saltychev@gmail.com.

## Declarations

### Clinical tools and procedures

This was a register-based study that utilized the data stored in electronic patient records.

## Abbreviations

PROM: Patient reported outcome measure
ODI: Oswestry Disability index
EFA: Exploratory Factor Analysis
CFA: Confirmatory Factor Analysis
