# Supplementary figures and images for: Reliability and validity of Oswestry Disability Index among patients undergoing lumbar spinal surgery
Source: BMC Surg. 2024 Jan 3;24:13. doi: 10.1186/s12893-023-02307-w (PMC10765861; doi:10.1186/s12893-023-02307-w)

Supplement 1. Factor analysis by surgery types - scree plots and path diagrams

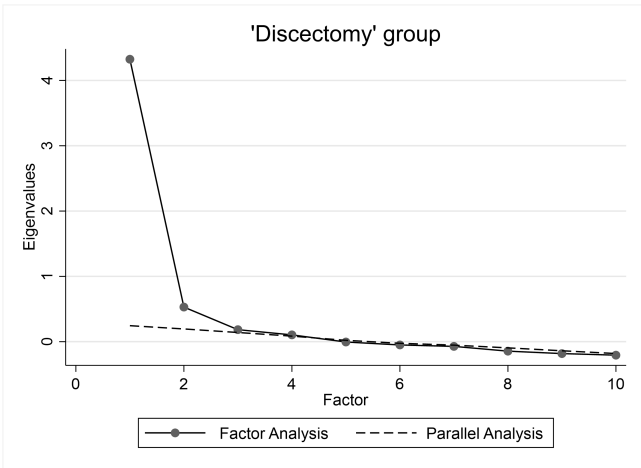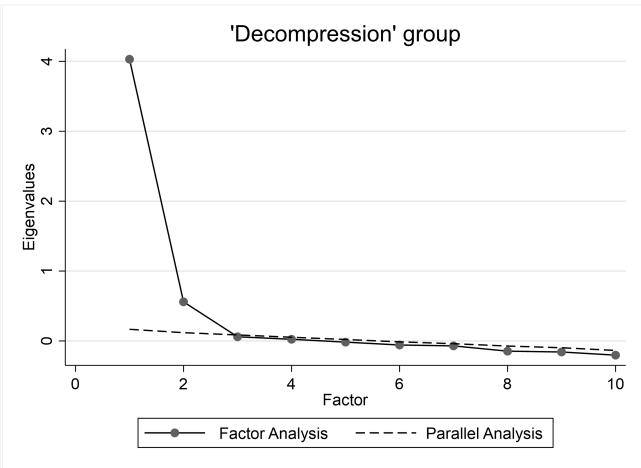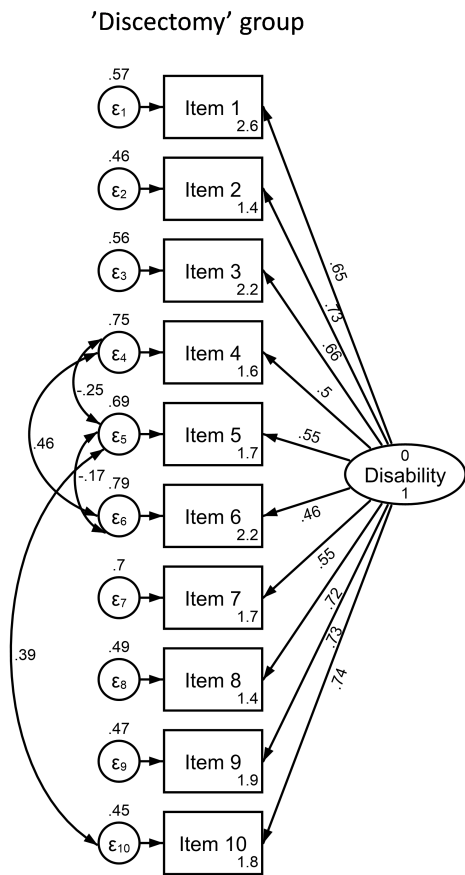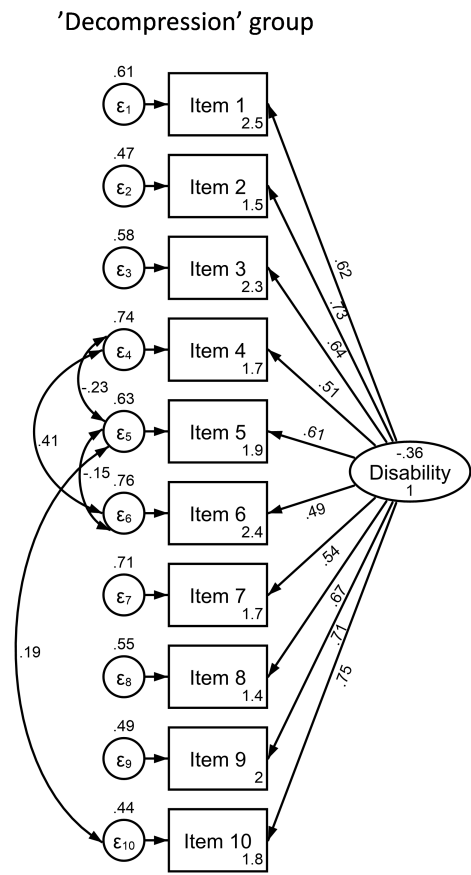

Supplement: Supplementary file 1 — Supplementary Material 1. Factor analysis by surgery types - scree plots and path diagrams [file 12893_2023_2307_MOESM1_ESM.pdf]
